# Commentary: Epidemiology, economics and the path to clean energy

**DOI:** 10.1093/ije/dyaa224

**Published:** 2020-12-08

**Authors:** Philip J Landrigan, Aaron Bernstein

**Affiliations:** 1 Program for Global Public Health and the Common Good, Boston College, Boston, MA, USA; 2 Harvard Medical School, Boston, MA, USA; 3 Division of General Pediatrics, Boston Children’s Hospital, Boston, MA, USA; 4 Center for Climate, Health and the Global Environment, Harvard T.H. Chan School of Public Health, Boston, MA, USA

For the first time in history, heat and electricity can be produced more cheaply today from wind and solar energy than from any fossil fuel in many parts of the world.^1^ This is an extraordinary paradigm shift in global energy production. It could not have been foreseen even 5 years ago. It offers us all the prospect of a reprieve from some of the worst consequences of global climate change.

Since the invention of the steam engine more than 200 years ago, fossil fuels have powered the world. Combustion of coal, oil and, most recently, natural gas, has provided the energy that made possible the Industrial Revolution, enabled the growth of modern urbanized societies and supported enormous advances in human health, well-being and longevity.

We now know, however, that these gains came at great cost. Reliance on fossil fuels has resulted in astounding amounts of pollution, enough to kill 9 million people worldwide in a year and stifle economic growth by 6.2%—or US$4.6 trillion per year.^2^ Even more damaging is the overwhelming contribution that our use of fossil fuels has made to global climate change^3^; 80% of the greenhouse gas emissions that drive climate change come from fossil fuels. The good life that so many of us in the world’s high-income countries experience today was achieved, in the words of the *Lancet* Commission on Planetary Health, by mortgaging humanity’s future and leaving future generations a legacy of disease, death and environmental degradation.^4^

Combustion of coal and oil continues to rise around the world, but now even many in the fossil fuel industry acknowledge that these fuels are no longer sustainable. Natural gas has thus been put forward as a better alternative. It is portrayed as a ‘bridge fuel’ between the more polluting fossil fuels of the past and clean, renewable energy.^5^^,^^6^

Production of natural gas has increased sharply. The USA, for example, extracted four times as much gas last year as it did two decades ago.^7^ This increase had been made possible by widespread adoption of ‘unconventional’ extraction methods that rely on hydraulic fracturing (‘fracking’). Fracking involves the high-pressure injection of large volumes of water, sand and proprietary chemicals deep underground to fracture rock beds and release trapped oil and gas. Fracking has resulted in a global glut of oil and gas at historically low prices.^7^

This abundance of inexpensive gas has prompted a vast expansion of fossil fuel infrastructure—wells, pipelines, compressor stations, ports and storage facilities—in countries around the world. Some 72 major gas pipelines and 82 liquefied natural gas terminals are currently under construction, with hundreds more proposed.^8^ The estimated cost of the gas terminals alone is US$1.3 trillion.^9^

The abundance of gas has led also rapid expansion in its use as a feedstock in plastic and chemical production.^10^ Gas is used today in the manufacture of an enormous array of products ranging from medications to baby bottles, clothing and fertilizer. Plastic manufacture is a major emerging market for the fossil fuel industry.

In almost all countries, expansion of the natural gas infrastructure is supported by taxpayer-supported subsidies and tax breaks given by governments to the fossil fuel industry.^11^ At the same time, governments allow the fossil fuel industry to externalize the health and environmental costs of the harms caused by gas extraction and transmission. These costs are thus borne by taxpayers and not included in the market price of gas.

Proponents of natural gas describe it as a cheap and abundant fuel that produces less air pollution and greenhouse gases than coal or oil per unit energy produced. But those claims overlook the emerging evidence documenting that gas is as dangerous, and possibly more dangerous, than other fossil fuels.^12^^,^^13^

Gas extraction is linked to contamination of ground and surface water, air pollution, noise and light pollution, ecological damage and earthquakes. Gas pipelines and storage facilities leak, catch fire and explode. Compressor stations, required to push gas through pipelines, emit carcinogenic chemicals such as benzene, 1,3-butadiene and formaldehyde. Gas combustion generates oxides of nitrogen and ammonia that can trigger asthma attacks and increase hospitalizations in people with chronic obstructive pulmonary disease. Perhaps most concerning is the main substance of natural gas: methane. Methane is a powerful greenhouse gas with a heat-trapping potential 30 times greater than that of carbon dioxide over a 100-year span and 85 times greater over a 20-year span. Of all fracked gas, 4% or more is lost to leakage and these releases appear to have contributed to recent, unprecedented increases in atmospheric methane.^14^

Claims that natural gas causes little harm to health were made before the emergence of any scientific data to the contrary.^13^ Now, a series of large, well-conducted epidemiological studies on the hazards of gas extraction and use have been published. These analyses focus largely on perinatal and paediatric outcomes, and they document multiple harms to children’s health in relation to gas development. These include elevated risks of preterm birth,^15–17^ low birthweight,^18^ decreased term birthweight^18^ and babies born small for gestational age.^18^ Two of these studies report positive trends between gas production volume and frequency of adverse birth outcomes.^17^^,^^18^ Emerging evidence suggests that exposure to gas extraction may increase risk for congenital heart defects and paediatric leukaemia.^19^^,^^20^

The sophisticated study by Mary Willis and her colleagues^21^ in this issue of the *International Journal of Epidemiology* adds to the growing literature on the hazards to children’s health of fracking. Willis and her colleagues combined information from two large datasets: (i) a database of Texas inpatient hospitalizations for the years 2000 to 2010 which recorded the residential address (zip code) of each child hospitalized with asthma; and (ii) a commercial database showing the geocoded location of each gas well in Texas, its production volume and its technology, conventional or unconventional. They found a consistent and statistically significant increase in risk of asthma hospitalization with increasing exposure to natural gas drilling. Risk increased with increasing production volume. Risks were similar for conventional and unconventional drilling.

Willis and her colleagues found that risk of asthma hospitalization was especially great among children exposed to fracking in low-income, predominantly minority communities. This finding is consistent with the large and growing environmental justice literature documenting time and again that the people among us who suffer most from extraction and use of fossil fuels are the weakest and most vulnerable.^22^

Growing recognition of the hazards of natural gas, coupled with the plummeting cost of producing electricity from renewable, force us to confront the question: why do we need gas?

The economic arguments against gas are powerful. Since 2010, the cost of producing electricity from solar cells has declined by 81% and from wind by 45%.^1^ These costs are expected to decrease still farther over the next 5 years, as ever more electricity is produced from wind and solar and advantages of scale are realized. At the same time, investment in renewables is increasing exponentially^23^ and in 2021 will for the first time exceed spending on oil and gas exploration.^24^

Finally, it must be noted that the current cost advantage of wind and solar power over gas would be even greater were it not for two factors that artificially distort energy markets: the government subsidies and tax breaks that depress the market price of natural gas,^11^ and the industry’s ability—with government permission—to externalize the costs of the health and environmental harms caused by gas.

Several paths can lead us away from the multiple harms to human and planetary health caused by natural gas and toward a clean energy future. Perhaps the most effective would be a requirement that the full costs of the damages caused by natural gas be included in its market price. Fifty years ago, the USA chose to take this path to control air pollution from coal-fired power plants. In consequence, the market price of electricity produced from coal rose significantly, coal-fired power plants became increasingly uneconomical, hundreds of thousands of lives were saved and millions of heart attacks, strokes, cancers and asthma attacks were prevented. Coal has become an economically foolish investment, and many coal-fired power plants are now stranded assets.^25^ The same would be true for natural gas today if we paid the true cost of its extraction and use.^9^

A second way to reduce reliance on gas is to end all subsidies and tax breaks for its production and transmission.^11^

At the same time as we curtail fossil fuel use and phase out its economic support system, we must invest generously in the communities that for generations have produced the coal, oil and gas that powered the world’s growth. These hard-working communities cannot be abandoned. They deserve sustained, thoughtful, multiyear investments in education, health care and infrastructure which will enable them to move forward and enhance their health and well-being.

Epidemiologists, physicians, nurses and health scientists are in a uniquely powerful position to contribute to the conversation about energy futures. Few other groups in our society enjoy such respect and trust—especially now—or are better equipped to educate policy makers and the public about the threats posed to human health by the continued use of gas and other fossil fuels. Just as the epidemiologists of a generation ago made critical contributions to reducing the threat of nuclear war, ^26^ epidemiologists today can speak truth to power and provide the leaders of our cities, states and countries the information that will empower them to take courageous actions to end our reliance on fossil fuels.

## Author contributions

Both authors provided equally to writing the original and subsequent drafts. Both approved the final version of the manuscript.
